# Explicit and Implicit Emotional Expression in Bulimia Nervosa in the Acute State and after Recovery

**DOI:** 10.1371/journal.pone.0101639

**Published:** 2014-07-02

**Authors:** Salomé Tárrega, Ana B. Fagundo, Susana Jiménez-Murcia, Roser Granero, Cristina Giner-Bartolomé, Laura Forcano, Isabel Sánchez, Juan José Santamaría, Maher Ben-Moussa, Nadia Magnenat-Thalmann, Dimitri Konstantas, Mikkel Lucas, Jeppe Nielsen, Richard G. A. Bults, Tony Lam, Theodoros Kostoulas, Nikos Fakotakis, Nadine Riesco, Ines Wolz, Josep Comín-Colet, Valentina Cardi, Janet Treasure, José Antonio Fernández-Formoso, José Manuel Menchón, Fernando Fernández-Aranda

**Affiliations:** 1 Departament de Psicobiologia i Metodologia de les Ciències de la Salut, Universitat Autònoma de Barcelona, Barcelona, Spain; 2 Ciber Fisiopatologia Obesidad y Nutrición (CIBERObn), Instituto de Salud Carlos III, Madrid, Spain; 3 Department of Psychiatry, Bellvitge University Hospital-IDIBELL, Barcelona, Spain; 4 Clinical Sciences Department, School of Medicine, University of Barcelona, Barcelona, Spain; 5 Faculty of Social Sciences and Economics, University of Geneva, Geneva, Switzerland; 6 Serious Game Interactive (SGI), Copenhagen, Denmark; 7 MobiHealth B.V., Enschede, The Netherlands; 8 NetUnion, Lausanne, Switzerland; 9 Wire Communications Laboratory, University of Patras, Patras, Greece; 10 Department of Cardiology, Institut Hospital del Mar d'Investigacions Mèdiques, Barcelona, Spain; 11 Eating Disorders Unit, Institute of Psychiatry, King's College, London, United Kingdom; 12 CIBER, Instituto de Salud Carlos III, Madrid, Spain; 13 CIBER Salud Mental (CIBERSAM), Instituto Salud Carlos III, Barcelona, Spain; University of Barcelona, Faculty of Biology, Spain

## Abstract

Expression of emotional state is considered to be a core facet of an individual's emotional competence. Emotional processing in BN has not been often studied and has not been considered from a broad perspective. This study aimed at examining the implicit and explicit emotional expression in BN patients, in the acute state and after recovery. Sixty-three female participants were included: 22 BN, 22 recovered BN (R-BN), and 19 healthy controls (HC). The clinical cases were drawn from consecutive admissions and diagnosed according to DSM-IV-TR diagnostic criteria. Self reported (explicit) emotional expression was measured with State-Trait Anger Expression Inventory-2, State-Trait Anxiety Inventory, and Symptom Check List-90 items-Revised. Emotional facial expression (implicit) was recorded by means of an integrated camera (by detecting Facial Feature Tracking), during a 20 minutes therapeutic video game. In the acute illness explicit emotional expression [anxiety (p<0.001) and anger (p<0.05)] was increased. In the recovered group this was decreased to an intermediate level between the acute illness and healthy controls [anxiety (p<0.001) and anger (p<0.05)]. In the implicit measurement of emotional expression patients with acute BN expressed more joy (p<0.001) and less anger (p<0.001) than both healthy controls and those in the recovered group. These findings suggest that there are differences in the implicit and explicit emotional processing in BN, which is significantly reduced after recovery, suggesting an improvement in emotional regulation.

## Introduction

Concerns about weight and shape were central to early maintenance models of BN [1] but over time these models have been extended to include problems in social emotional functioning [2]. A model using the SPAARS framework (schematic, propositional, analogical and associative representation systems) of emotional processing posits that eating disorder symptoms develop as a means of managing negative emotions such as anger by redirecting the emotion onto the self and body, in the form of self-disgust/shame [3].

Systematic reviews of the domains of social-emotional processing in people with eating disorders found substantial difficulties in different domains, including emotion recognition and emotion regulation [4]. A meta analysis showed that people with bulimia nervosa had particular problems with social evaluative aspects of functioning (negative self evaluation, higher sensitivity to rank related issues, etc.) [5]. Experimental implicit tasks in people with eating disorders find negative self esteem and vigilance towards critical and dominant faces [6]. This may explain why anger is suppressed as suggested by SPAARS model as anger expression is less tolerated in those with subservient positions [7].

However, emotional expression, both implicit and explicit, has been less often studied in people with BN. Thereby, explicit emotion regulation processes require a conscious effort to be initiated and completed, some monitoring during implementation, and a certain degree of awareness (e.g. answering questions on a questionnaire). Implicit processes are automatic, can happen without insight or awareness, and do not need monitoring in order to be completed (e.g. facial expressions, attentional bias…) [8]. In a preliminary pilot study we found that patients with BN showed significantly less facial expression of anger/frustration than controls while playing a therapeutic video game [9]. On the other hand, expressions of joy were higher than healthy controls in people with BN which contrasted with the lower level of expression of positive facial affect in patients with anorexia nervosa. However the implicit vigilance towards facial signals of criticism and dominance was associated with early adversity [6].

In order to examine for possible state or trait effects, the aim of the study was to examine the implicit emotional expression (by measuring facial expression in response to a therapeutic video game) and explicit emotional expression (measured by self report of anxiety and anger) in BN patients, in both acute and recovered state compared with healthy controls. We hypothesised that patients with BN would show lower emotional regulation functioning, expressed by higher levels of positive emotion and reduced anger than healthy controls, which might improve after remission.

## Materials and Methods

### Subjects

The study was carried out according to the latest version of the Declaration of Helsinki and was approved by the Ethics Committee of the Bellvitge University Hospital (Spain). Written informed consent was obtained from all participants.

The study was conducted between May 2011 and June 2013. Sixty-three women participants were included, distributed in three independent groups: 22 BN patients in acute state, 22 BN patients in remission state and 19 healthy controls. Clinical cases were diagnosed according to DSM-IV-TR diagnostic criteria [10], by means of structured clinical interview for DSM-IV Axis I disorders (SCID-I) [11], conducted by experienced psychologists and psychiatrists. The criteria to be included as recovered patients were having a minimum of 12 (consecutive) weeks being abstinent from bingeing and purging (laxatives and/or vomiting). As described in previous studies [12], [13], the inclusion criteria was an age between 18 and 45 years. The exclusion criteria were: primary psychiatric or neurological disorders (e.g. psychotic disorders, bipolar disorders, major depressive disorders, substance abuse-dependence disorders, etc.) or active pharmacological therapy that can interfere with the game performance; current or lifetime diagnosis of behavioural technological addictions. Patients were consecutive referrals for assessment and outpatient treatment at our Hospital.

Participants' characteristics are shown in Table 1, No statistical differences between groups were found in neither age nor marital status, but a larger percentage of the healthy control group had a high education level. Both clinical groups (BN in acute state and BN in remission state) reported: a non-significantly different mean duration of eating disorders illness, and non-significantly different mean Body mass index. BN patients reported mean weekly frequency of bingeing of 4.1 (SD = 3.8) and a mean weekly frequency of vomiting of 7.2 (SD = 15.5). None of the BN patients had a lifetime diagnosis of AN. The healthy control cohort included 19 volunteers from the same catchment area. They did not receive any financial compensation for their time.

**Table 1:** Sociodemographic variables.

	BN	R-BN	HC	
	(N = 22)	(N = 22)	(N = 19)	*p*
Age (years); *mean (SD)*	28.9 (7.8)	27.2 (8.6)	29.4 (8.1)	0.661
Marital status; *% Single-widow*	71.4	72.7	63.2	0.674
* Married-coupled*	19.0	13.6	31.6	
* Divorced-separated*	9.5	13.6	5.3	
Education level *Primary or less*	15.0	15.0	0.0	0.002
* Secondary*	70.0	70.0	31.6	
* University*	15.0	15.0	68.4	
ED duration (years); *mean (SD)*	11.7 (7.8)	8.9 (6.7)	---	0.300
Body mass index (kg/m^2^); *mean (SD)*	23.7 (3.0)	24.9 (4.1)	---	0.313

BN: Bulimia Nervosa; R-BN: Recovered BN; HC: Healthy Controls; SD: Standard deviation;

ED: Eating disorder; BMI: body mass index. --- Not available for this cohort.

### Measures

#### Explicit Emotional expression measures

State-Trait Anxiety Inventory (STAI) [14], [15]: is a self-report questionnaire that includes 40 items on the basis of a 1-4 response scale, to evaluate the temporary condition of “state anxiety”(S) (20 items) and the more long-standing quality of “trait anxiety”(T) (20 items). The set of questions value feelings of anxiety and depression in the areas of worry, tension and apprehension. The STAI was validated in Spanish population with Cronbach's alpha coefficients ranging between 0.90 and 0.94 [16].

Symptom Check List-90 items-Revised (SCL-90-R) [17]: is a multidimensional self-report assessment measure for a broad range of psychological problems/symptoms. It contains 90 items structured in nine primary symptom dimensions: Somatization, Obsession- Compulsion, Interpersonal Sensitivity, Depression, Anxiety, Hostility, Phobic Anxiety, Paranoid Ideation, and Psychoticism. In addition, the questionnaire can produce three global scores: a global severity index (GSI, which measures global psychological distress), a positive symptom distress index (PSDI, a measure of the intensity of symptoms) and a positive symptom total (PST, which reports the total self-reported symptoms). Only the Anxiety subscale was used in this study. This questionnaire has been extensively validated in a Spanish population, obtaining adequate psychometrical values [18]. This scale has been validated in a Spanish population, obtaining a mean internal consistency of 0.75 (Coefficient alpha) [19].

State-Trait Anger Expression Inventory 2 (STAXI-2) [20] is a 44-item self-report instrument that examines the experience and expression of anger. This instrument was validated in a Spanish population with Cronbach's alpha coefficients ranging between 0.63 and 0.95 [21]. The Spanish version of the STAXI-2 comprises 49 items [21]. It entails six scales: (1) State Anger; (2) Trait Anger; (3) Anger Control (including two subscales: a) Anger Control-Out and b) Anger Control-In); (4) Anger Expression-In; (5) Anger Expression-Out and (6) Anger Expression Index, which provides a general index of the expression of anger (derived from the Anger Expression-In, the Anger Expression-Out and the Anger Control scales). Items are rated on four-point Likert scales assessing either the intensity of the angry feelings or the frequency with which anger is experienced, expressed, suppressed, or controlled.

#### Implicit Emotional expression measure

Facial recognition software: as described in previous studies [9], [12], this facial affect recognition software was designed and developed for this specific PlayMancer Platform. The facial expression of the patient during the video game performance is detected by an integrated camera and processed by the facial tracking component. For this experiment, we used anger and joy emotions as outcome measures. To calibrate the facial emotion recognition software, several previous experiments were conducted, for a more detailed description of the method see this study [9]. The measure provided by this tool is the total time (in seconds) that the patient is facially expressing a particular emotion throughout the duration of the entire video game session.

### Procedure

For both clinical groups and healthy controls, experienced psychologists/psychiatrists conducted face-to-face structured interviews. Participants completed the self-report questionnaires (SCL-90-R, STAI and STAXI-2). For the BN patients in acute state, the video game session took place before starting cognitive behavioural therapy (CBT). For the BN patients in remission state, the session was recorded in a follow-up session after finishing the standard CBT program [13].

#### The video game intervention

A detailed description of the *Island* Video game is available in [12]. It has been used as an add-on therapeutic tool for ED with promising results [13]. The overall goal is to improve self-control and also to learn how to regulate arousal and reactivity to negative situations, such as frustration, anxiety and time pressure. Biofeedback and a focus on breathing to produce relaxation are used to train this form of emotional regulation [9]. The level of game difficulty is adjusted in a closed feedback loop; higher levels of undesired emotional and/or physiological reactions are coupled with greater difficulty in attaining the end goals. The performance in each VG session was collected during 20 min. Three minutes of relaxing music were played before and after the VG session. The VG consists of three mini-games: (1) The Face of Cronos: The player has to climb up a cliff in which obstacles appear, depending on the arousal of the player (based on biofeedback). This mini-game trains planning and decision making; (2) Treasures of the Sea: A virtual swimming game in which the player has to collect different objects and fishes while conserving their oxygen supply. This trains visuospatial abilities, visual working memory and decision making. High arousal makes the task more difficult; (3) Sign of the Magupta: A relaxation game in which the player connects a constellation of stars through breathing control. Slow deep breathing allows the connections between stars to form [13].

### Statistical analysis

Analyses were carried out with SPSS20 for Windows. The comparison of emotion measures between diagnostic groups was carried out with Poisson regression, a log-linear model that uses the logarithm as the link function and the Poisson distribution function and becomes useful for count data. Analysis of Variance (ANOVA) tested the mean differences in psychological measures and performance measure among the groups. The effect size was valued with the 95% confidence interval for the contrasts and with the Cohen's d coefficient (good effect sizes were considered for |d|>0.50). Analyses were adjusted by the SCL-Depression-score.

## Results

### Measures of implicit emotional expression: facial expression

Table 2 shows the descriptive for the facial expression of joy and anger (in seconds) and the comparison between groups. Mean scores showed that BN patients expressed joy during the longest time and anger during the shortest time, whereas healthy controls expressed joy during shortest time and anger for the longest. All the pair wise comparisons achieved significant results, except for the post-hoc comparison of joy between Recovered-BN and healthy controls.

**Table 2:** Association between group and implicit emotional expression (facial expression) measures.

	Descriptives				*Comparison between diagnostic conditions*								
	Mean (standard deviation)				BN vs. R-BN			BN vs. HC			R-BN vs. HC		
	BN (N = 22)	R-BN (N = 22)	HC (N = 19)	*p*	MD	*p*	95%CI	MD	*p*	95%CI	MD	*p*	95%CI
Joy (seconds)	951.9 (7.7)	874.4 (7.4)	882.9 (7.7)	<0.001	77.5	<.001	(56.6; 98.4)	68.9	<0.001	(47.6; 90.3)	−8.56	0.422	(−29.4; 12.3)
Anger (seconds)	45.0 (1.7)	103.1 (2.5)	192.0 (3.6)	<0.001	−58.1	<.001	(−64.1; −52.2)	−147.0	<0.001	(−154.7; −139.3)	−88.9	<0.001	(−97.5; −80.3)

Results for comparisons obtained with ANOVA procedures. In bold Significant contrasts (.05 level).

HC: Healthy Controls; BN: Bulimia Nervosa; R-BN: recovered BN. MD: mean difference.

In order to control for effects of playing success on the expression of emotions, the outcome of the diving performance on the mini-game “treasures of the sea” was calculated as a number of errors (number of times out of breath) divided by the minutes playing the diving mini-game. No statistical differences were found between groups (p = 0.538) [BN: 0.28(0.26); R-BN: 0.23(0.10); HC: 0.22(0.10)].

**Figure 1:**
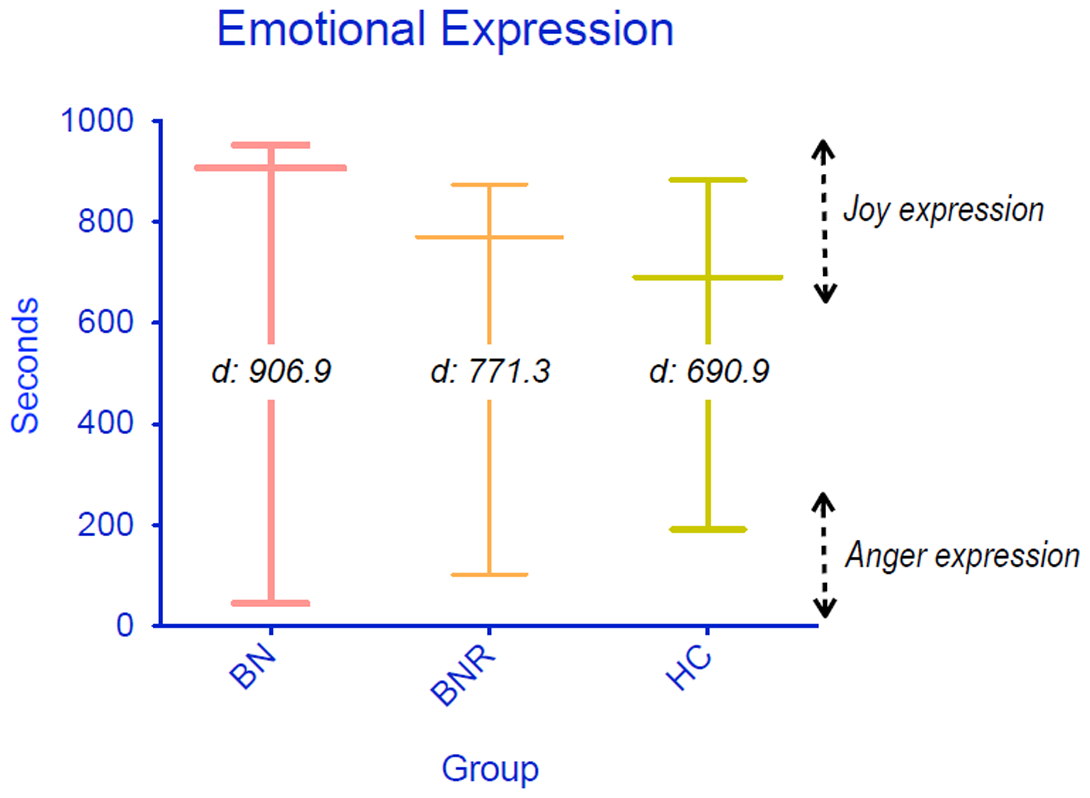
Average difference between positive and negative implicit emotional expression among the groups.

Figure 1 shows the differences between joy and anger expression. Each vertical line begins in the anger mean score (45.0 for BN, 103.1 for R-BN and 192.0 for HC) and ends in the joy mean measure (951.9, 874.4 and 882.9 for each group); the longest horizontal-line for each group represents the mean difference between joy and anger expressions (951.9–45.0 = 906.9 for BN, 874.4–103.1 = 771.3 for R-BN and 882.9–192.0 = 690.9 for HC). As shown in Figure 1, the average difference between expression of positive and negative own implicit emotion was lower in recovered BN and healthy controls than in acute BN (p<0.001).

### Measures of explicit emotional expression: anxiety and anger

Table 3 shows the ANOVA models comparing the mean scores registered in the SCL-90-R-anxiety scale, STAI-state and trait scales, and the STAXI-state and trait scales. As a rule for all measures, BN patients achieved the highest mean scores and HC the lowest. All pair-wise comparisons between these two diagnostic conditions achieved significant differences with high effect sizes (|d|>0.80). BN-recovered group showed intermediate scores compared with BN and healthy controls. The pair-wise comparisons between BN- recovered and BN achieved statistical differences for SCL-90-R-anxiety and STAI. The comparison of BN- recovered with healthy controls achieved also significant and relevant differences for SCL-90-R-anxiety, STAI-trait and STAXI-state.

**Table 3:** Association between group and explicit emotional expression (anxiety, anger and impulsivity) measures.

	Mean (standard deviation)			Comparison between diagnostic conditions								
	BN	R-BN	HC		BN vs. R-BN			BN vs. HC			R-BN vs. HC		
	(N = 22)	(N = 22)	(N = 19)	*p*	MD	(95% CI)	|d|	MD	(95% CI)	|d|	MD	(95% CI)	|d|
SCL-90R: anxiety	1.81 (0.91)	1.19 (0.93)	0.30 (0.29)	<0.001	0.62*	(0.01; 1.23)	0.68	1.51*	(0.86; 2.16)	2.23	0.89*	(0.22; 1.55)	1.28
STAI													
State	30.7 (9.31)	20.5 (13.3)	16.4 (11.8)	0.001	10.2*	(1.28; 19.0)	0.89	14.3*	(4.69; 24.0)	1.35	4.17	(−5.26; 13.6)	0.33
Trait	37.5 (8.20)	25.4 (15.7)	13.1 (5.76)	<0.001	12.1*	(3.53; 20.6)	0.97	24.4*	(15.1; 33.7)	3.44	12.3*	(3.18; 21.4)	1.04
STAXI													
Anger State	22.0 (9.62)	18.7 (8.16)	15.5 (0.61)	0.027	3.26	(−2.54; 9.07)	0.37	6.53*	(0.72; 12.3)	0.96	3.26	(−2.62; 9.15)	0.56
Anger Trait	24.6 (7.46)	21.4 (8.25)	18.3 (5.15)	0.028	3.13	(−2.47; 8.73)	0.40	6.29*	(0.68; 11.9)	0.98	3.16	(−2.52; 8.83)	0.46
Anger Index	32.5 (12.1)	26.9 (12.4)	24.3 (7.39)	0.057	5.63	(−2.90; 14.2)	0.46	8.26	(−0.27; 16.8)	0.82	2.63	(−6.11; 11.4)	0.26
Expression Out	12.2 (3.76)	11.0 (3.65)	10.8 (2.67)	0.394	1.19	(−1.48; 3.86)	0.32	1.35	(−1.32; 4.01)	0.41	0.16	(−2.57; 2.89)	0.05
Expression In	14.8 (4.49)	16.4 (5.63)	18.4 (3.77)	0.047	0.87	(−2.19; 3.94)	0.20	3.09*	(0.02; 6.15)	0.84	2.21	(−0.93; 5.35)	0.62
Control Out	14.8 (4.49)	16.4 (5.63)	18.4 (3.77)	0.065	−1.61	(−5.27; 2.05)	0.32	−3.56	(−7.22; 0.10)	0.86	−1.95	(−5.70; 1.81)	0.41
Control In	11.7 (3.04)	15.1 (3.50)	14.3 (3.86)	0.009	−3.34*	(−6.05; −0.63)	1.02	−2.60	(−5.31; 0.11)	0.75	0.74	(−2.04; 3.51)	0.20

Results for comparisons obtained with ANOVA procedures. In bold, medium to high effect size (|*d*|>0.50). *Significant contrasts (.05 level).

BN: Bulimia Nervosa; R-BN: Recovered BN; HC: Healthy Controls. MD: mean difference. |*d*|: Cohen's d.

## Discussion

The aims of the study were to examine implicit aspects of emotional regulation by measuring facial expression in response to a therapeutic video game (Islands), and explicit aspects of emotional reactivity (i.e. anger and anxiety), measured by self-reported questionnaires, in both acute and recovered states of BN patients. We were able to confirm our first hypotheses in that patients with BN did show higher levels of implicit positive emotion and reduced implicit anger than healthy controls, and BN recovered patients had an intermediate response. In contrast to the reduced implicit expression of anger through facial expression, in the questionnaires asking explicitly for emotional expression, patients with BN reported higher levels of state and trait anger and anxiety. These findings suggest an opposite profile in the explicit self reported emotions and implicit emotional expression in BN patients, which is visibly corrected in the recovered group.

The results point to emotional dysregulation in BN patients, with a selective reduction in the facial emotional expression of anger and elevated levels of explicit anger. This suggests a specific strategy of suppressing anger. These findings fit with the SPAARS model, as it is as if anger is appraised as ego-dystonic. It is possible that expressing anger has perhaps been linked to rejection from others, a form of emotional invalidation which may have been learned through classical conditioning. Therefore it appears that people with BN have learned to suppress anger which may have an outlet via restriction/bingeing–vomiting. These results are in line with those suggesting that ED patients have inadequate anger expression and deficits in dealing with anger and frustration [22], [23]. There is evidence suggesting that ED patients experience negative emotions as threatening, dangerous and unacceptable and their expression as a sign of weakness and reason for social rejection [24]–[26].

This lack of concordance between implicit and explicit negative emotional expression has been noted to occur when emotional suppression is used as a form of emotional regulation [27], [28]. The recurrent use of emotional suppression in the long term produces reduced control of emotion, problems in interpersonal functioning, and higher depressive symptomatology [29]. The mental effort required to suppress facial reactivity may make the individual less responsive to the emotional cues of the person they are interacting with and this may disrupt social functioning. This may contribute to the lack of trust and problems with conflict noted in bulimia nervosa [30]. Neuroimaging studies have also demonstrated that suppression is associated with higher activation in the amygdale and insula [31], [32], and in regions implicated in cognitive control, namely prefrontal and anterior cingulated cortices [33], [34]. These findings demonstrate that emotional suppression is attained at a higher physiological, psychological and interpersonal cost [32], [35].

The BN patients also showed greater levels of facial expression of positive emotion and yet described themselves as more anxious than the control group. However, in the recovered group there is a decrease in the level of discordance between this implicit and explicit emotions, which might indicate that after remission BN patients exhibit a more authentic emotional response [24], [36], as high levels of positive emotional expression in BN patients might be used as a means of gaining acceptance and avoiding rejection [37]. Finally, the convergence between anxiety and emotional expression in BN patients is in agreement with studies showing that anxiety is associated with the inability to control emotional responses [38], [39]. Neuroimaging studies also support this hypothesis, and it has been suggested that bigger decreases in anxiety are related to higher ventro-medial prefrontal cortex activity, a cerebral region involved in emotional regulation [40]–[42].

The study adds to earlier work on emotional expression in ED, which had found less facial expression in AN during negative and positive film clips and attenuated verbal emotional expression in AN during an emotion talk task, but not in BN [43], [44]. Since the outcome variable in this study was verbal expression, the results of the study at hand amends to the existing research by suggesting that facial emotional expression, which may be less susceptible to cognitive control, does show disturbances in patients with acute BN. Since the video game can be seen as emotionally neutral, it would be interesting to assess the facial emotional expression of BN patients during positive and negative film clips.

This study has several important strengths, primarily the inclusion of both, acutely ill BN patients and BN patients after remission. Although emotional regulation has been previously studied in BN [13], [22] this is, to the best of our knowledge, the first time that the implicit and explicit emotional expression has been described in BN patients in the acute and recovered state. However, the results of this study should be interpreted in the context of some limitations. First, the sample was relatively small, so the results should be interpreted with caution. Second, only women were included, considering that it has been estimated that only 10–15% of people with ED are male [45]. Future studies focusing on the emotional regulation of male BN patients are desirable. As a third point it has to be mentioned that the explicit measure only included negative emotions (anger and anxiety), while implicit facial expression was assessed as well in the positive (joy) as in the negative (anger) valence. Thus, no conclusion on the coherence between implicit and explicit expression of the positive emotion joy could be drawn. The possibility that BN patients actually felt more emotions that are positive cannot be ruled out. In addition, the assessment of implicit and explicit emotional expression was taken during different activities, more precisely explicit emotions were assessed during the completion of questionnaires, implicit emotions were assessed as reaction towards a video game. Game-playing could have influenced the emotional state of participants, resulting in an increase of positive emotions and a decrease of negative emotions in the patient group. Still, there aren't any conclusive hints to support these assumptions, since game success was controlled for.

Finally yet importantly, the question of the relationship between emotion regulation and emotional expression needs to be addressed. From the results of this study it cannot be confirmed if the differences in emotional expressivity and the discordance between implicit and explicit emotional expression are due to difficulties in emotion regulation and emotional awareness, because other explaining factors or intervening variables (such as previous emotional experiences and specific personality traits) cannot be excluded. It is cognisable that other factors related to the psychopathology of eating disorders account for differences in facial emotional expression. Since sleep disturbances and its interaction with altered neurotransmitter function (e.g. leptin and orexin) [46]–[48] were not assessed in this study, an influence of these variables is possible. Thus, it is not clear if obtained results are due to improvements in emotional abilities or to improvements in before mentioned factors. Although it is probable that the patients' emotion regulation capacities have improved with treatment and that the more authentic expression of emotions can be explained by this, future studies should address these issues.

In summary, this study emphasizes the importance of developing treatments targeting more effective strategies for enhancing the regulation of emotions as well as their adequate recognition and authentic expression in BN patients. Future neuropsychological and neuroimaging studies should focus on the emotional profile of these patients, in order to shed more light on these multifaceted constructs.
